# PPARγ Mediates Transdifferentiation of CX3CR1^+^-Derived Cells into Adipocytes

**DOI:** 10.3390/ijms27062917

**Published:** 2026-03-23

**Authors:** Yong-Feng Yang, Cheng-Chao Ruan, Yu Lei

**Affiliations:** Department of Physiology and Pathophysiology, State Key Laboratory of Medical Neurobiology, School of Basic Medical Sciences, Fudan University, Shanghai 200032, China; yongfengyang0986@163.com

**Keywords:** CX3CR1, transdifferentiation, adipocyte, PPARγ

## Abstract

Transdifferentiation of one cell type into another occurs under normal physiological conditions. Adipose tissue is an important metabolic and endocrine organ involved in the onset and progression of various diseases. Previous studies have shown that fibroblasts can transdifferentiate into adipocytes. Here, we demonstrate that CX3CR1-derived cells can also transdifferentiate into adipocytes. Additionally, RFP^+^ SVF cells and mature adipocytes were identified in different adipose tissues of *Cx3cr1^cre^: Rosa26^Td^* mice. Cold exposure enhances the adipogenic transdifferentiation of RFP^+^ cells, whereas a high-fat diet (HFD) inhibits this process. Mechanistically, we found that PPARγ regulates transdifferentiation, suggesting its role in the differentiation of CX3CR1-derived cells into adipocytes, thus offering new insights into the origin of adipocytes in the body.

## 1. Introduction

Irregular diets and lifestyles can lead to excessive accumulation of adipose tissue, contributing to metabolic diseases, such as obesity, hypertension, cardiovascular disease, atherosclerosis, and so on. Adipose tissue is a highly heterogeneous endocrine organ that stores energy in lipid form. Adipose tissue is classified into brown adipose tissue (BAT), beige adipose tissue (beige AT), and white adipose tissue (WAT), which contain various cell types, including adipocytes, preadipocytes, mesenchymal stem cells, immune cells, endothelial cells, and sympathetic nerve cells. In rodents, BAT is located in the interscapular dorsal region and is mainly composed of brown adipocytes, which are rich in small lipid droplets and mitochondria for thermogenesis and anti-inflammatory functions. In humans, BAT is present in the subclavicular region of infants and adults, especially during cold exposure [1,2,3]. WAT is located in both visceral and subcutaneous areas and consists of white adipocytes, which store excess energy as large lipid droplets [4,5]. Beige adipose tissue, a unique type of adipose tissue, has features of both WAT and BAT. In response to cold or exercise, brown adipocytes can emerge within white adipose tissue, enhancing lipolysis and thermogenesis [6,7]. Adipose tissue plays a critical role in regulating body health and the development of obesity-related diseases and cardiovascular diseases.

CX3CR1, a chemokine receptor, was initially known to be expressed in microglia in the brain but has since been identified in macrophages of the heart, gut, and adipose tissue. CX3CR1^+^ macrophages primarily engage in anti-inflammatory or pro-inflammatory roles [5,6]. Recently, it has been shown that CX3CR1 regulates obesity-related inflammatory and metabolic responses in adipose tissue [7,8]. Obesity induces senescence of adipose-derived stem cells (ASC) in visceral adipose tissue (VAT), and high expression of CX3CR1 can delay ASC senescence, supporting metabolic adaptation [7]. Additionally, CX3CR1 is also implicated in the repair of intestinal defects through stromal vascular fraction (SVF) treatment [9].

The stromal vascular fraction (SVF) of adipose tissue comprises adipose-derived stem cells, mesenchymal progenitor/stem cells, endothelial progenitor cells, pericytes, fibroblasts, and immune cells (T cells, macrophages) [8,9,10]. Additionally, PDGFRα serves as a marker for adipocyte precursors. Adipocytes primarily differentiate from adipocyte precursors [11] and mesenchymal stem cells within the SVF [12]. Transdifferentiation refers to the process of one specialized cell type transforming into another [13,14,15]. Adipocyte transdifferentiation is essential for maintaining metabolic homeostasis. Previous studies indicate that transcription factors regulate adipose transdifferentiation. Specifically, FoxO1 promotes the transdifferentiation of beige adipocytes into white adipocytes by regulating the expression of Tgfβ1, inhibiting the expression of UCP1 protein, and increasing lipid droplet accumulation in adipocytes [16]. Furthermore, peroxisome proliferator-activated receptor (PPARγ), PR domain containing 16 (PRDM16), and early B-cell factor 2 (EBF2) are involved in adipose tissue browning and regulate the transformation of white adipocytes into brown adipocytes [17]. Previous studies also showed that growth factors participate in adipocyte transdifferentiation. Moreover, growth factors, including VEGF, regulate adipocyte transdifferentiation, as VEGF promotes the transformation of white adipocytes into beige adipocytes and vice versa [18]. Studies have also shown that primary keloid myofibroblasts promote the protein expression of PPARγ and C/EBPα and increase lipid droplet formation upon botulinum toxin A treatment, leading to fibroblast transdifferentiation into adipocyte-like cells [19]. Besides fibroblasts, myoblasts also promote the transcription of adipogenic genes and the accumulation of lipid droplets through all-trans retinoic acid. This process upregulates PPARγ levels. PPARγ agonists like rosiglitazone promote adipogenesis, whereas PPARγ inhibitors such as BADGE reduce PPARγ levels and suppress transdifferentiation [20]. Unsaturated fatty acids also upregulate PPARγ and lipid droplet formation in human cancer cell lines, which transdifferentiate into adipocyte-like cells [21]. Heparanase 2 (Hpa2) deficiency can lead to the transdifferentiation of pancreatic acinar cells into adipocytes [22]. Furthermore, compounds like apigenin promote the transdifferentiation of white adipocytes into brown adipocytes through VEGF-PRDM16 signaling and angiogenesis [23]. Small-molecule compounds also induce adipose transdifferentiation. Small-molecule mixtures such as Repsox, VPA, and TTNPB have also been shown to induce transdifferentiation of fibroblasts into adipocyte-like cells by significantly upregulating adipose-related gene transcription and protein levels [24].

However, it remains unclear whether immune cells can transdifferentiate into adipocytes. In this study, we observed that RFP^+^ SVF cells and RFP^+^ mature adipocytes were present in different adipose tissues of *Cx3cr1^cre^: Rosa26^Td^* mice. The RFP^+^ SVF cells include CX3CR1^+^ immune cells, transdifferentiated PDGFRα^+^ adipocyte precursor cells (previously expressing CX3CR1), and CX3CR1^+^PDGFRα^+^ transitional cells. Additionally, cold exposure enhanced the adipogenic differentiation of RFP^+^ cells, which was inhibited in HFD mouse models. We further demonstrated that PPARγ regulates the adipogenic differentiation of RFP^+^ cells, providing a further understanding of the origin of adipocytes.

## 2. Results

### 2.1. RFP^+^SVF Cells Are Present in Adipose Tissue of Cx3cr1^cre^: Rosa26^Td^ Mice

**Figure 1:**
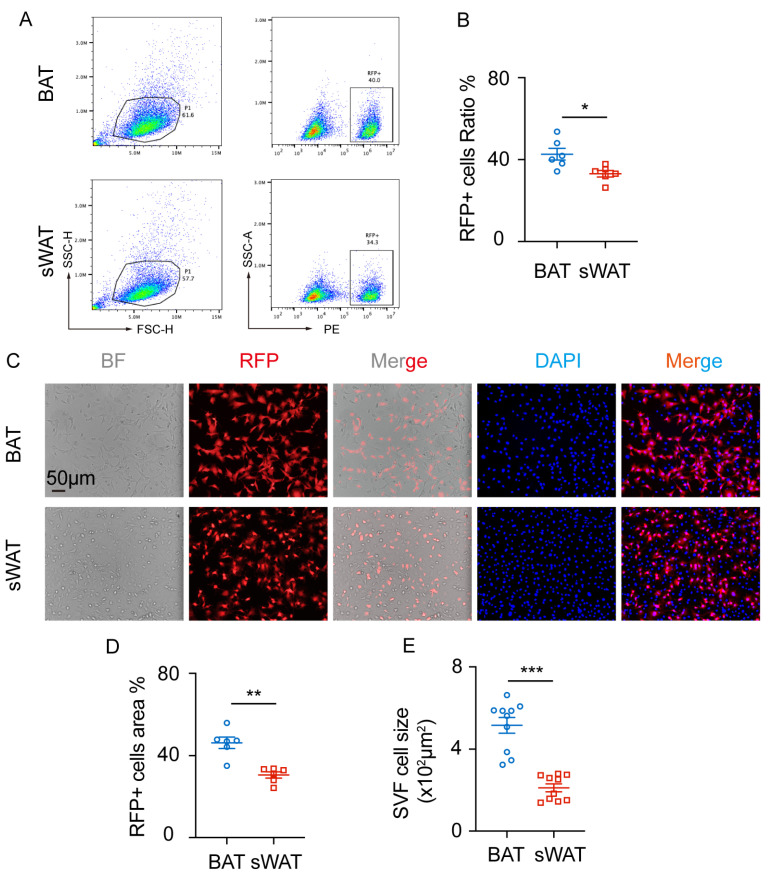
RFP^+^SVF cells in adipose tissue of *Cx3cr1^cre^: Rosa26^Td^* mice. (**A**) Representative images of RFP^+^ cells in BAT and sWAT. (**B**) Quantification of RFP^+^ cell ratio in BAT and sWAT. *n* = 6 per group. (**C**) Representative images of SVF cells from BAT and sWAT. (**D**) Quantification of RFP^+^ cell area ratio of SVF cells in BAT and sWAT. *n* = 6 per group. (**E**) Quantification of SVF cells’ size in BAT and sWAT. *n* = 10 cells per group. Scale bars: 50 μm. Values represent mean ± SEM. Student’s *t*-tests (**B**,**D**,**E**), *** *p* < 0.001, ** *p* < 0.01, * *p* < 0.05.

To explore whether immune cells also transdifferentiate into adipocytes, we constructed *Cx3cr1^cre^: Rosa26^Td^* mice and analyzed SVF cells isolated from BAT and sWAT. Flow cytometric analysis revealed that the proportions of RFP^+^ cells in BAT and sWAT were 42.62 ± 2.877% and 33.10 ± 1.588%, respectively, with a statistically significant difference between the two tissues (Figure 1A,B).

Next, SVF cells from adipose tissue were cultured in vitro for further analysis. The uncultured SVF cells predominantly consist of adipose-derived stem cells, mesenchymal progenitor/stem cells, endothelial progenitor cells, pericytes, fibroblasts, and immune cells. After culturing, the SVF cells mainly consist of adipose-derived stem cells. Spontaneous fluorescence analysis detected RFP-positive cells in the SVF of BAT and sWAT (Figure 1C). The proportions of RFP^+^ cells in BAT and sWAT were 46.25 ± 2.757% and 30.56 ± 1.524%, respectively (Figure 1D). In addition, the SVF cells in BAT were larger than those in sWAT (Figure 1E). Subsequently, we investigated the potential of RFP^+^SVF cells to differentiate into mature adipocytes.

### 2.2. Transdifferentiation of RFP^+^SVF Cells into Mature Adipocytes

**Figure 2:**
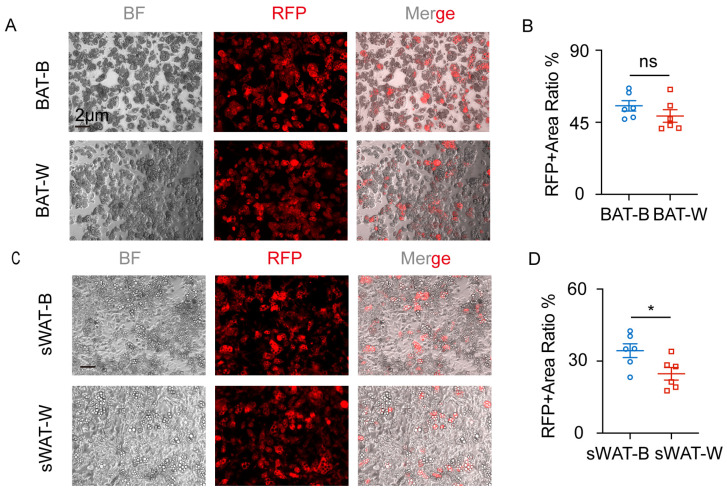
RFP^+^SVF cells transdifferentiate into adipocytes in BAT and sWAT of *Cx3cr1^cre^: Rosa26^Td^* mice. (**A**) Representative images of RFP^+^SVF cells differentiated into adipocytes in BAT of *Cx3cr1^cre^: Rosa26^Td^* mice. (**B**) Quantification of RFP^+^ area ratio in BAT of *Cx3cr1^cre^: Rosa26^Td^* mice. (**C**) Representative images of RFP^+^SVF cells differentiated into adipocytes in sWAT of *Cx3cr1^cre^: Rosa26^Td^* mice. (**D**) Quantification of RFP^+^ area ratio in sWAT of *Cx3cr1^cre^: Rosa26^Td^* mice. *n* = 6 per group. Scale bars: 2 μm. Values represent mean ± SEM. Student’s *t*-tests (**B**,**D**), * *p* < 0.05, ns = no significant.

RFP^+^ SVF cells from BAT and sWAT were induced into brown and white adipocytes using an adipogenic induction experiment. Our results showed that RFP^+^SVF cells in BAT differentiated into brown adipocytes and white adipocytes (Figure 2A), with no significant difference in differentiation capacity (Figure 2B). Meanwhile, RFP^+^SVF cells in sWAT differentiated into brown adipocytes and white adipocytes (Figure 2C). Notably, the differentiation potential was stronger toward brown adipocytes compared to white adipocytes in sWAT. (Figure 2D).

We also observed that RFP^+^ SVF cells in *Cx3cr1^cre^: Rosa26^Td^* mice were transdifferentiated into adipocytes in vitro. Next, we explored whether this phenomenon also exists in the adipose tissue of *Cx3cr1^cre^: Rosa26^Td^* mice in vivo.

### 2.3. Cold Exposure Promotes Lipogenesis of RFP^+^ Cells from Different Adipose Tissues in Cx3cr1^cre^: Rosa26^Td^ Mice

**Figure 3:**
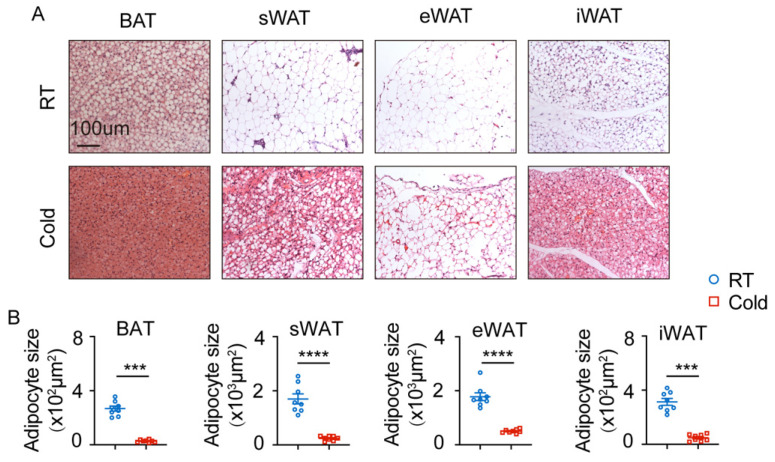
Cold exposure promotes lipogenesis of adipocytes in BAT, sWAT, eWAT, and iWAT of *Cx3cr1^cre^: Rosa26^Td^* mice. (**A**) H&E staining of BAT, sWAT, eWAT, and iWAT under cold exposure. (**B**) Quantification of adipocyte size of BAT, sWAT, eWAT, and iWAT under cold exposure. *n* = 8 cells per group. Scale bars: 100 μm. Values represent mean ± SEM. Student’s *t*-tests (**B**), **** *p* < 0.0001, *** *p* < 0.001.

The cold exposure model was employed to assess lipolysis, browning of adipose tissue, and heat production of brown adipose tissue. The *Cx3cr1^cre^: Rosa26^Td^* mice were then exposed to room temperature (RT) and cold exposure for further analysis. Hematoxylin and eosin (HE) staining revealed that cold exposure significantly reduced the size of adipocytes in BAT, sWAT, eWAT, and iWAT in mice (Figure 3A,B).

**Figure 4:**
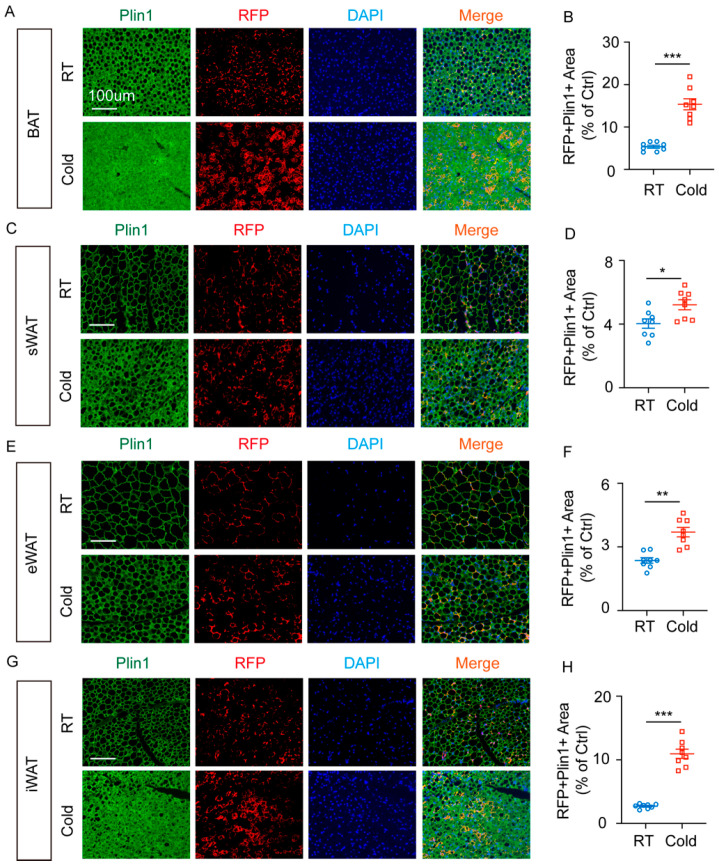
Cold exposure promotes lipogenesis of RFP^+^ cells in BAT, sWAT, eWAT, and iWAT of *Cx3cr1^cre^: Rosa26^Td^* mice. (**A**) Representative images of Plin1 expression of RFP^+^ cells in BAT of *Cx3cr1^cre^: Rosa26^Td^* mice under RT and cold exposure. (**B**) Quantification of RFP^+^ Plin1^+^ area in BAT of *Cx3cr1^cre^: Rosa26^Td^* mice under RT and cold exposure. (**C**) Representative images of Plin1 expression of RFP^+^ cells in sWAT of *Cx3cr1^cre^: Rosa26^Td^* mice under RT and cold exposure. (**D**) Quantification of RFP^+^ Plin1^+^ area in sWAT of *Cx3cr1^cre^: Rosa26^Td^* mice under RT and cold exposure. (**E**) Representative images of Plin1 expression of RFP^+^ cells in eWAT of *Cx3cr1^cre^: Rosa26^Td^* mice under RT and cold exposure. (**F**) Quantification of RFP^+^ Plin1^+^ area in eWAT of *Cx3cr1^cre^: Rosa26^Td^* mice under RT and cold exposure. (**G**) Representative images of Plin1 expression of RFP^+^ cells in iWAT of *Cx3cr1^cre^: Rosa26^Td^* mice under RT and cold exposure. (**H**) Quantification of RFP^+^ Plin1^+^ area in iWAT of *Cx3cr1^cre^: Rosa26^Td^* mice under RT and cold exposure. *n* = 8 per group. Scale bars: 100 μm. Values represent mean ± SEM. Student’s *t*-tests (**B**,**D**,**F**,**H**), *** *p* < 0.001, ** *p* < 0.01, * *p* < 0.05.

Additionally, Plin1 (Perilipin-1), a marker of mature adipocytes, was analyzed through immunofluorescence staining. Cold exposure promoted lipogenic differentiation of RFP^+^ cells in BAT (Figure 4A,B). Further examination of sWAT (Figure 4C) showed similar promotion of lipogenic differentiation in RFP^+^ cells (Figure 4D). Similarly, cold exposure induced lipogenic differentiation of RFP^+^ cells in eWAT (Figure 4E,F) and iWAT (Figure 4G,H).

RFP^+^ mature adipocytes were identified across various adipose tissues in *Cx3cr1^cre^: Rosa26^Td^* mice. Cold exposure further promoted adipogenic differentiation in RFP^+^ cells.

### 2.4. Cold Exposure Promotes CX3CR1 and PDGFRα Expression of RFP^+^ Cells in BAT and sWAT of Cx3cr1^cre^: Rosa26^Td^ Mice

The proportion of RFP^+^ SVF cells was higher in BAT and sWAT of *Cx3cr1^cre^: Rosa26^Td^* mice compared to the proportion of tissue immune cells under normal physiological conditions. The Cre recombinase in *Cx3cr1^cre^: Rosa26^Td^* mice is constitutively expressed, maintaining RFP expression even after the loss of the CX3CR1 marker. To investigate the real-time expression of CX3CR1 in RFP^+^ cells, mice were exposed to cold exposure.

**Figure 5:**
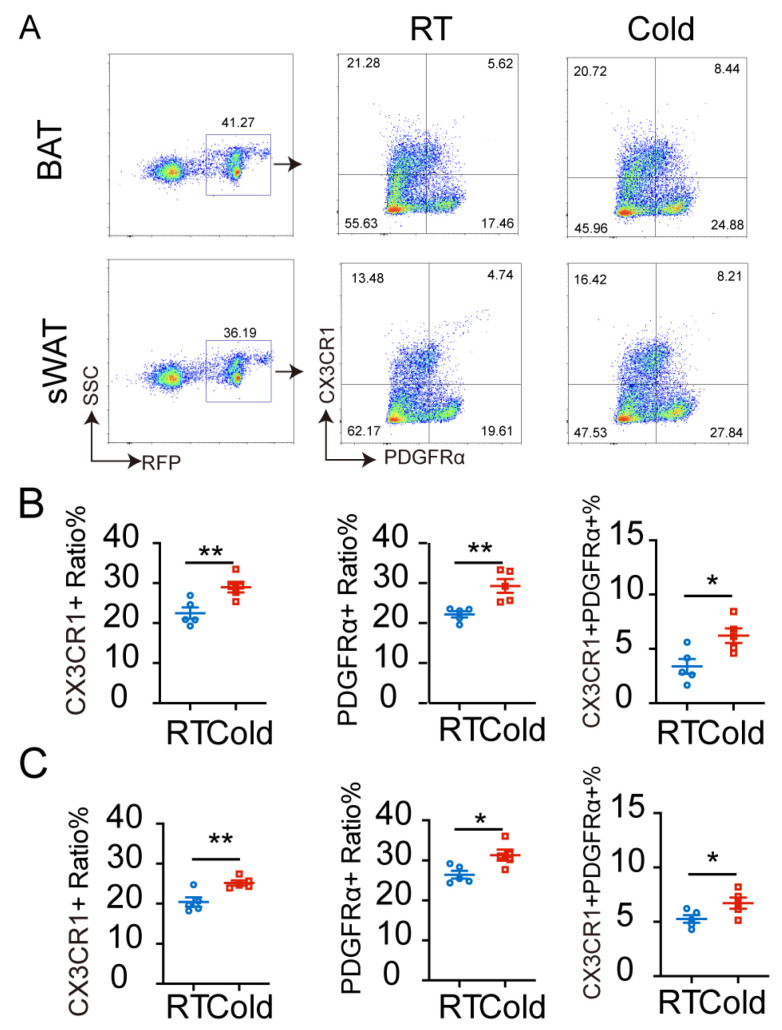
Cold exposure promotes CX3CR1 and PDGFR*α* expression of RFP^+^ cells in BAT and sWAT of *Cx3cr1^cre^: Rosa26^Td^* mice. (**A**) Flow cytometric analysis of expression of CX3CR1 and PDGFR*α* by RFP^+^ SVF cells in BAT and sWAT of *Cx3cr1^cre^: Rosa26^Td^* mice under RT and cold exposure. Quantification of CX3CR1^+^ and PDGFR*α*^+^ ratio by RFP^+^ SVF cells in BAT (**B**) and sWAT (**C**) of *Cx3cr1^cre^: Rosa26^Td^* mice under RT and cold exposure. *n* = 5 per group. Values represent mean ± SEM. Student’s *t*-tests (**B**,**C**), ** *p* < 0.01, * *p* < 0.05.

Furthermore, flow cytometry was used to analyze changes in adipocyte precursor markers (PDGFRα) and immune cell-specific markers (CX3CR1) to characterize the RFP^+^ cells. SVF cells were isolated from BAT and sWAT of *Cx3cr1^cre^: Rosa26^Td^* mice under RT and cold exposure. Flow cytometric analysis of CX3CR1 and PDGFRα in RFP^+^ SVF cells showed that RFP^+^ SVF cells could express CX3CR1 and PDGFRα. The proportion of CX3CR1^+^ cells among RFP^+^ SVF cells in BAT was 22.5 ± 1.408%, the proportion of PDGFRα^+^ cells was 22.18 ± 0.7753%, and the proportion of CX3CR1^+^PDGFRα^+^ cells was 3.388 ± 0.6861%. The proportion of CX3CR1^+^ cells among RFP^+^SVF cells in sWAT was 20.42 ± 1.155%, the proportion of PDGFRα^+^ cells was 26.41 ± 0.9751%, and the proportion of CX3CR1^+^PDGFRα^+^ cells was 5.258 ± 0.3526%. This indicates that RFP^+^ SVF cells include CX3CR1^+^ immune cells, transdifferentiated PDGFRα^+^ adipocyte precursor cells (previously expressing CX3CR1), and transitional cells (CX3CR1^+^PDGFRα^+^) capable of transdifferentiating into PDGFRα^+^ adipocyte precursor cells. Notably, cold exposure increased the proportions of CX3CR1^+^ or PDGFRα^+^ cells within RFP^+^ SVF cells. A subset of these cells is the origin of the proportion of CX3CR1^+^PDGFRα^+^ double-positive cells, whose proportion is significantly elevated under cold exposure (Figure 5A–C). These results suggest that the increased PDGFRα^+^ adipogenic precursor cells under cold exposure mainly originate from the transition of CX3CR1^+^ cells differentiating into PDGFRα^+^ to form CX3CR1^+^PDGFRα^+^ transitional cells, which then differentiate into PDGFRα^+^ adipogenic precursor cells.

### 2.5. Obesity Inhibits RFP^+^ Cell Lipogenesis in Different Adipose Tissues in Cx3cr1^cre^: Rosa26^Td^ Mice

To further explore the adipogenic differentiation potential of RFP^+^ cells in *Cx3cr1^cre^: Rosa26^Td^* mice, we utilized obesity models (mice fed a high-fat diet (HFD)). HFD promotes adipocyte hypertrophy and induces chronic inflammation. The *Cx3cr1^cre^: Rosa26^Td^* mice were fed either a normal diet or HFD to potential the lipid formation in RFP^+^ cells by immunostaining of different adipose tissues.

**Figure 6:**
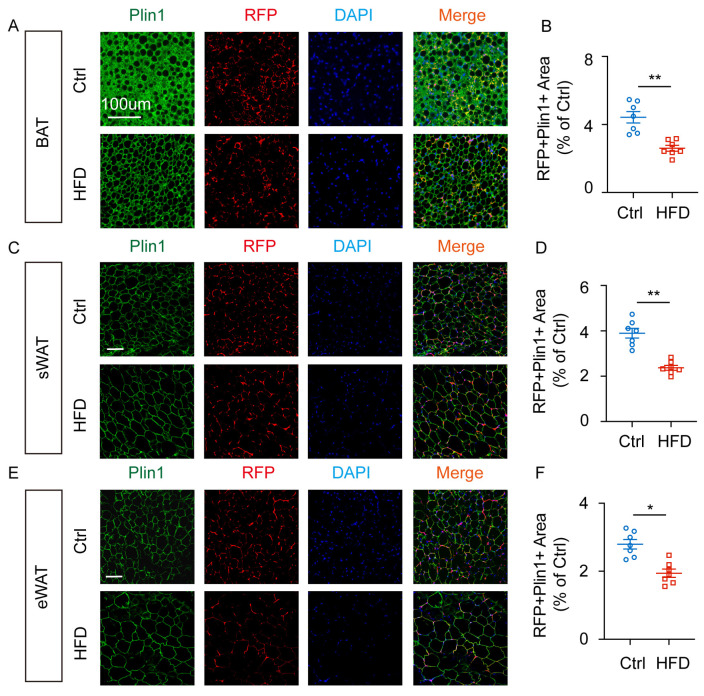
HFD inhibits lipogenesis of RFP^+^ cells in BAT, sWAT, and eWAT of *Cx3cr1^cre^: Rosa26^Td^* mice. (**A**) Representative images of Plin1 expression in BAT of *Cx3cr1^cre^: Rosa26^Td^* mice under HFD. (**B**) Quantification of RFP^+^ Plin1^+^ area in BAT of *Cx3cr1^cre^: Rosa26^Td^* mice under HFD. (**C**) Representative images of Plin1 expression in sWAT of *Cx3cr1^cre^: Rosa26^Td^* mice under HFD. (**D**) Quantification of RFP^+^ Plin1^+^ area in BAT of *Cx3cr1^cre^: Rosa26^Td^* mice under HFD. (**E**) Representative images of Plin1 expression in eWAT of *Cx3cr1^cre^: Rosa26^Td^* mice under HFD. (**F**) Quantification of RFP^+^ Plin1^+^ area in BAT of *Cx3cr1^cre^: Rosa26^Td^* mice under HFD. *n* = 7 per group. Scale bars: 100 μm. Values represent mean ± SEM. Student’s *t*-tests (**B**,**D**,**F**), ** *p* < 0.01. * *p* < 0.05.

Immunofluorescence staining revealed that HFD inhibited the lipogenic differentiation of RFP^+^ cells in mouse BAT (Figure 6A,B). Similarly, HFD also inhibited the lipogenic differentiation in RFP^+^ cells in sWAT (Figure 6C,D) and eWAT (Figure 6E,F). These results indicate that HFD may increase the inflammation, promoting the accumulation of CX3CR1^+^ immune cells and inhibiting the adipogenic differentiation of RFP^+^ cells.

### 2.6. PPARγ Mediates the Adipogenic Differentiation of RFP^+^SVF Cells in Adipose Tissue of Cx3cr1^cre^: Rosa26^Td^ Mice

Although RFP^+^ cells in *Cx3cr1^cre^: Rosa26^Td^* mice can transdifferentiate into adipocytes, the specific regulatory mechanisms remain unclear. Previous studies have shown that various transcription factors are involved in the adipogenesis process. Specifically, PPARγ is involved in the browning of adipose tissue and regulates the transdifferentiation of white adipocytes into brown adipocytes [17]. To investigate whether PPARγ regulates lipogenic differentiation of RFP^+^ cells, in vitro cell experiments were conducted.

**Figure 7:**
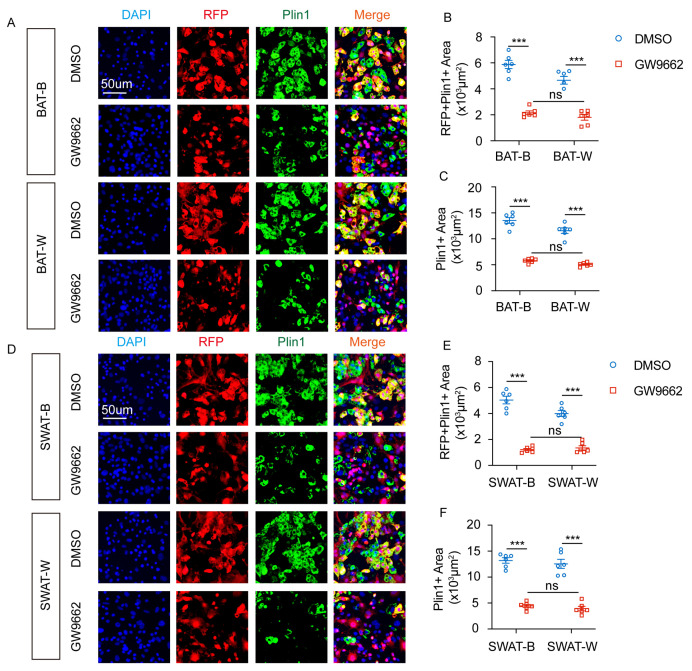
GW9662 inhibits the lipogenic differentiation of RFP^+^SVF cells in BAT and sWAT of *Cx3cr1^cre^: Rosa26^Td^* mice. (**A**) Representative images of Plin1 expression of RFP^+^ SVF cells differentiated into brown adipocyte (BAT-B) and white adipocyte (BAT-W) after DMSO or GW9662 treatment. (**B**) Quantification of RFP^+^ Plin1^+^ area of BAT-B and BAT-W after DMSO or GW9662 treatment. (**C**) Quantification of Plin1^+^ area of BAT-B and BAT-W after DMSO or GW9662 treatment. (**D**) Representative images of Plin1 expression of RFP^+^ SVF cells differentiated into brown adipocyte (sWAT-B) and white adipocyte (sWAT-W) after DMSO or GW9662 treatment. (**E**) Quantification of RFP^+^ Plin1^+^ area of sWAT-B and sWAT-W after DMSO or GW9662 treatment. (**F**) Quantification of Plin1^+^ area of sWAT-B and sWAT-W after DMSO or GW9662 treatment. *n* = 6 per group. Scale bars: 50 μm. Values represent mean ± SEM. Two-way ANOVA (**B**,**C**,**E**,**F**), *** *p* < 0.001, ns = no significant.

SVF cells were isolated from BAT and sWAT of *Cx3cr1^cre^: Rosa26^Td^* mice, then differentiated into brown adipocytes and white adipocytes. The cells were treated with the PPARγ antagonist GW9662 to evaluate its impact on adipogenic differentiation of RFP^+^ cells. GW9662 inhibited Plin1 expression in BAT-B and BAT-W adipocytes (Figure 7A,B) and the double-positive area of RFP^+^Plin1^+^ (Figure 7C). Additionally, GW9662 inhibited the expression of Plin1 in sWAT-B and sWAT-W adipocytes (Figure 7D,E) and the co-labeled positive area of RFP^+^Plin1^+^ (Figure 7F).

In vitro cell experiments showed that PPARγ inhibition reduced the differentiation efficiency of SVF cells in BAT and sWAT, thereby limiting lipogenic differentiation of RFP^+^SVF cells. These results indicate that GW9662 inhibits lipid formation by blocking transdifferentiation of RFP^+^ cells.

In summary, RFP^+^ SVF cells and RFP^+^ mature adipocytes are identified in different adipose tissues of *Cx3cr1^cre^: Rosa26^Td^* mice. RFP^+^ SVF cells include transdifferentiated PDGFRα^+^ adipocyte precursor cells and CX3CR1^+^PDGFRα^+^ transitional cells. Cold exposure promotes adipogenic differentiation, whereas HFD inhibits adipogenic differentiation through PPARγ.

## 3. Discussion

Adipose tissue is highly responsive to the metabolic state of the body. Most studies on the origin of adipose cells focus on the transdifferentiation between white adipocytes and brown adipocytes, as well as the transdifferentiation of fibrocytes, pancreatic acinar cells, and even cancer cells. Immune cells within adipose tissue primarily regulate the inflammatory response of adipose tissue. Notably, immune cells and adipose cells have distinct functions. Herein, RFP^+^ cells of Cx3cr1^+^ in the adipose tissue of *Cx3cr1^cre^: Rosa26^Td^* mice were shown to transdifferentiate into adipocytes. CX3CR1, a G-protein-coupled receptor, is predominantly expressed in monocytes, macrophages, and T cells [25]. The results indicate that CX3CR1^+^ cells are initially expressed in immune cells in the early stage, but during later stages of development, these cells may lose CX3CR1 expression while retaining the RFP imprint and transdifferentiate into adipocytes. This is the first study to indicate that CX3CR1^+^ immune cells can transdifferentiate into adipocytes.

Furthermore, the proportion of RFP^+^SVF cells in both BAT and sWAT of *Cx3cr1^cre^: Rosa26^Td^* mice was found to exceed 30%, which is higher than the proportion of immune cells in adipose tissue [26,27]. This suggests that RFP^+^ cells represent a conventional immune cell population together with other RFP^+^ cell populations. Flow cytometric analysis revealed that RFP^+^SVF cells include transdifferentiated PDGFRα^+^ adipocyte precursor cells and CX3CR1^+^PDGFRα^+^ transitional cells, which are capable of further transdifferentiating into mature adipocytes. CX3CR1^+^PDGFRα^+^ double-positive cells indicate that CX3CR1^+^ cells have the potential to transdifferentiate into mature adipocytes, and the PDGFRα^+^ adipocyte precursor cells may be derived from post-expressed CX3CR1 cells. Additionally, lipid induction experiments showed that RFP^+^SVF cells could differentiate into both brown adipocytes and white adipocytes, suggesting that RFP^+^ SVF cells can develop into mature adipocytes.

Further findings revealed the presence of RFP^+^ mature adipose cells in BAT, sWAT, eWAT, and iWAT of *Cx3cr1^cre^: Rosa26^Td^* mice. Also, cold exposure significantly enhanced lipogenesis of RFP-positive cells, while HFD led to the opposite results. PPARγ is a key transcription factor associated with lipid formation. Herein, the PPARγ antagonist GW9662 inhibited the differentiation of RFP^+^SVF cells into adipocytes, indicating that PPARγ regulates transdifferentiation of SVF cells into adipocytes.

In conclusion, this study identifies a new origin for adipocyte transdifferentiation, bridging CX3CR1^+^ immune cell development with adipocyte formation.

## 4. Materials and Methods

### 4.1. Animals

The animal experiments were approved by the Animal Management and Use Committee of the School of Basic Medicine of Fudan University. The animals were treated following the regulations of the laboratory animal management of Shanghai Medical College of Fudan University. The animals were housed in a pathogen-free SPF environment in the Laboratory Animal Service Center of Fudan University. The feeding environment was maintained on a 12-h light/dark cycle with ad libitum access to chow diet and water. *C57BL/6* mice were sourced from GemPharmatech company. *Cx3cr1^cre^* mice were sourced from Jackson Laboratory (Bar Harbor, ME, USA, JAX 025524). *Rosa26^Td^* mice were obtained from Jackson Laboratory (JAX 007914). *Cx3cr1^cre^: Rosa26^Td^* mice were bred by mating *Cx3cr1^cre^* mice and *Rosa26^Td^* mice.

### 4.2. Flow Cytometric Analysis

*Cx3cr1^cre^: Rosa26^Td^* mice were perfused with 1 × PBS. BAT and sWAT were excised, cut into small pieces (volume: 0.2 cm^2^), and digested with collagenase type II (2 mg/mL in DMEM/F12 medium) at 37 °C for 30 min while shaking. The digestion was stopped with complete medium (DMEM/F12 with 10% FBS and 1% penicillin/streptomycin). The digested samples were filtered through 40 μm strainers, centrifuged at 4 °C for 500× *g* for 5 min. The upper grease and liquid were discarded, and the precipitates were suspended in 1 × PBS +1%FBS solution. The suspended cells were incubated with Zombie Aqua™ dye (BioLegend, San Diego, CA, USA, Cat# 423101, 1:100), anti-PDGFR-α antibody (BioLegend, Cat# 135916, 1:100), and anti-CX3CR1 antibody (BioLegend, San Diego, CA, USA, Cat# 149029, 1:100) in the dark for 30 min. Then, the suspended cells were washed three times with 1 × PBS, centrifuged at 4 °C for 500× *g* for 5 min. Finally, the cells were suspended in 1 × PBS +1%FBS solution. The fluorescence of the RFP and antibody was analyzed using NovoExpress software version 1.6.0.

### 4.3. SVF Isolation

Stromal vascular fraction (SVF) was isolated after removing mature adipocytes from adipose tissue. SVF mainly contains adipose-derived stem cells, hematopoietic stem cells, endothelial cells, and immune cells [28]. Adipose tissue was digested and filtered, and then the single-cell suspension was centrifuged and counted. The cells were seeded in a well plate with complete medium and incubated at 37 °C for 2 days. The cells were washed with 1 × PBS to remove the mixed cells covered in the upper layer, and only the bottom adherent SVF cells were retained. Complete medium was replaced every two days.

### 4.4. SVF Adipogenic Differentiation and Inhibition Experiment

SVF cells were differentiated into mature brown and white adipocytes using some small molecular compounds after culturing for two days [29].

For mature brown adipocyte differentiation, SVF cells (60% confluence) were treated with complete medium supplemented with insulin (Meilunbio, Dalian, China, Cat# MB3848, 10 μg/mL), IBMX (Sigma, Shanghai, China, Cat# I7018, 0.5 mM), dexamethasone (Sigma, Cat# D4902, 1 μM), triiodothyronine (Sigma, Cat# T-074, 50 nM), and rosiglitazone (Sigma, Cat# R2408, 1 μM) for 2 days. IBMX and dexamethasone were then specifically removed from the medium after the cell morphology had significantly changed. The cells were differentiated into mature brown adipocytes with the formation of lipid droplets for 6–8 days. The medium was replaced every two days.

For mature white adipocyte differentiation, SVF cells (60% confluence) were treated with a complete medium supplemented with insulin (10 μg/mL), IBMX (0.5 mM), and dexamethasone (1 μM) for 2 days. Similarly, IBMX and dexamethasone were removed from the medium after the cell morphology had significantly changed. The cells were differentiated into mature white adipocytes with the formation of lipid droplets for 6–8 days. The medium was replaced every two days.

For the inhibition experiment, GW9662 (MedChemExpress (MCE), Shanghai, China, Cat# HY-16578, 5 μM) was added to the specific medium from day 3 to day 10–12. The control group was treated with 0.1% DMSO.

### 4.5. Cold Exposure

The *Cx3cr1^cre^: Rosa26^Td^* mice were housed at 10 °C for two weeks with ad libitum access to chow diet and water. Correspondingly, the *Cx3cr1^cre^: Rosa26^Td^* control mice were kept at room temperature (22 °C).

### 4.6. HFD Model

The *Cx3cr1^cre^: Rosa26^Td^* mice were fed a control diet or a 60% HFD (Dyets, Wuxi, China, Cat# HF60) for 12 weeks, with ad libitum access to chow diet and water.

### 4.7. H&E Staining

Adipose tissue was fixed with 4% PFA and dehydrated with 30%, 50% and 75% alcohol, followed by paraffin embedding. The paraffin sections were dewaxed with xylene and ethanol, stained with hematoxylin dye solution and eosin dye solution, and sealed with neutral resin. Images were obtained using Carl Zeiss Axio Imager M2 microscope (Carl Zeiss, Oberkochen, Germany).

### 4.8. Immunofluorescent Staining

The sections underwent antigen repair in sodium citrate for 15 min in a 95 °C water bath. The sections were then permeated and blocked with 0.3% Triton X-100 and 5% BSA at room temperature for 1 h, washed twice with 1 × PBST (5 min for every time), and incubated with the following primary antibodies at 4 °C overnight: Anti-Plin1 antibody (Abcam, Cambridge, UK, Cat# ab61682, 1:300), Anti- RFP antibody (ROCKLAND, Pottstown, PA, USA, Cat# 600-401-379, Cat# 200-301-379, 1:300), Anti-CX3CR1 antibody (Abcam, Cat# ab308613, 1:200). The sections were washed thrice with 1 × PBST (5 min for every time), and incubated with secondary antibodies in the dark for 1 h: Alexa Fluor^TM^488 donkey anti-goat IgG (H + L) (Thermo Fisher Scientific, Waltham, MA, USA, Cat# A11055 1:1000), Alexa Fluor^TM^594 donkey anti-rabbit IgG (H + L) (Thermo Fisher Scientific, Cat# A21207, 1:1000). The sections were washed thrice with 1 × PBST (5 min for every time) and incubated with DAPI (1:5000) at room temperature for 10 min. Finally, the sections were washed thrice with 1 × PBST (5 min each time). The slices were sealed with an anti-fluorescence quenching tablet. Images were captured at 20× magnification using a Carl Zeiss Axio Imager M2 microscope and a Leica TCS SP8 confocal microscope (Leica Microsystems, Wetzlar, Germany).

For SVF cell-induced lipogenesis, the samples were fixed with 4% PFA for 15 min, and washed twice with 1 × PBST (5 min for every time), then immunostained through the above permeable sealing and subsequent steps.

### 4.9. Statistical Analysis

Data are presented as means ± SEM. Values of *p* < 0.05 were considered to indicate a statistically significant difference. Statistical significance was determined using an unpaired Student’s *t*-test for two-group comparisons. Two-way ANOVA followed by Tukey’s test was used for multiple comparison analysis when there were two experimental factors. All the statistical analysis methods were indicated in the corresponding figure legends.

## Data Availability

The data presented in this study are available from the corresponding author on request.
